# Heat Transfer and Fluid Flow Characteristics of Microchannel with Oval-Shaped Micro Pin Fins

**DOI:** 10.3390/e23111482

**Published:** 2021-11-09

**Authors:** Yuting Jia, Jianwei Huang, Jingtao Wang, Hongwei Li

**Affiliations:** School of Energy and Power Engineering, Northeast Electric Power University, Jilin 132012, China; jyt@neepu.edu.cn (Y.J.); wjt219@gmail.com (J.H.); lihongwei@neepu.edu.cn (H.L.)

**Keywords:** microchannel, oval-shaped micro pin fins, heat transfer enhancement, fluid flow, structural design

## Abstract

A novel microchannel heat sink with oval-shaped micro pin fins (MOPF) is proposed and the characteristics of fluid flow and heat transfer are studied numerically for Reynolds number (*Re*) ranging from 157 to 668. In order to study the influence of geometry on flow and heat transfer characteristics, three non-dimensional variables are defined, such as the fin axial length ratio (*α*), width ratio (*β*), and height ratio (*γ*). The thermal enhancement factor (*η*) is adopted as an evaluation criterion to evaluate the best comprehensive thermal-hydraulic performance of MOPF. Results indicate that the oval-shaped pin fins in the microchannel can effectively prevent the rise of heat surface temperature along the flow direction, which improves the temperature distribution uniformity. In addition, results show that for the studied Reynolds number range and microchannel geometries in this paper, the thermal enhancement factor *η* increases firstly and then decreases with the increase of *α* and *β*. In addition, except for *Re* = 157, *η* decreases first and then increases with the increase of the fin height ratio *γ*. The thermal enhancement factor for MOPF with *α* = 4, *β* = 0.3, and *γ* = 0.5 achieves 1.56 at *Re* = 668. The results can provide a theoretical basis for the design of a microchannel heat exchanger.

## 1. Introduction

With the increasing advanced technology in energy, electronics, aerospace, medicine, chemical industry, and other fields, the corresponding equipment also gradually develop in the direction of high-power, high performance, and miniaturization, such as large solid laser array, high-power LED lighting equipment, high integration microelectronics devices, etc. These electronic devices usually have a small heat transfer surface but a high-heat flux rate [1,2]. This fact leads to high operating temperatures, which can significantly reduce the reliability of components and shorten their life. Therefore, it is necessary to develop new technologies that will be able to dissipate high heat fluxes.

Several heat dissipation methods, such as microchannel heat sink, micro refrigerator, micro heat pipe, and other micro-jet array [3], have been applied to solve the problem of thermal management. Among them, microchannel heat sink (MCHS) has been widely concerned because of its larger heat transfer area, better heat transfer performance, and easy packing. However, because of the increasing heat generation per unit volume of next generation electronic devices, traditional MCHS with simple structure can hardly meet the heat dissipation requirements. Therefore, it becomes particularly important to design more effective MCHS with stronger heat dissipation capability [4].

In the past two decades, a lot of investigations in improving the heat transfer performance of MCHS were published. The influence of the cross-sectional shape of the MCHSs on the flow and heat transfer performance, such as circular, triangular, and trapezoidal, have been studied by many researchers [5,6]. Subsequently, researchers devoted to investigate various techniques to promote heat transfer performance further. The influence of MCHS flow passage, such as wavy [7,8], zig-zag [9] and convergent-divergent [10], on the characteristics of flow and heat transfer have been studied by some authors. Moreover, the flow disruption techniques have been introduced to improve the thermal performance of MCHS, for example, ribs and groove structures [11,12,13], reentrant cavities [14], roughness surfaces [15], and MCHS combined with secondary oblique channels [16,17]. In addition, nanofluids have opened the doors towards the enhancement of microchannel thermal applications performance [18]. He and Yan [19] wrote a comprehensive review to describe the various heat transfer enhancement methods in a microchannel.

In addition to all that, it has been confirmed that inclusion of pin fin in micro heat sinks can significantly increase the overall heat transfer effectiveness. Vasilev et al. [20] compared the heat transfer performance of MCHS without pin fins and MCHS with cylindrical pin fins by computational simulation. Different structure sizes of pin fins, such as diameter, spacing, and height, in MCHSs were performed, and the results showed that the fluid disturbance was increased because of the placing of pin fins in the flow channels, which enhance the thermal performance of MCHS. For the purpose of improving the temperature uniformity of the cooled device, a micro-pin-fin heat sink with variable density was proposed by Vilarrubí et al. [21]. The results showed that the convection thermal resistance was decreased and the better surface temperature uniform was displayed by using increasing density of pin-fins along the flow direction. Prajapati et al. [22] studied numerically the heat transfer and fluid flow behavior of rectangular parallel microchannel heat sinks with varying fin heights. It has been observed that pen microchannel heat sinks have a greater advantage than the completely closed heat sink. Ventola et al. [23] proposed a diamond-shaped micro-protruded patterns heat sink and experimentally investigated the thermal fluid dynamics features. The geometrical parameters of diamond shaped fins were also studied and optimized, which revealed to have a significant influence on convective heat transfer. Wang et al. [24] proposed an optimal origami fin shape to enhance the forced and free convection heat transfer of the heat sink.

From the abovementioned review, it can be illustrated that the micro pin fin within the heat sink can obviously improve the thermal performance of cooling devices. Meanwhile, the heat transfer and pressure drop are directly dependent on the pin fin’s shape and their positions within the heat sink. In our preliminary work, a microchannel combined with cone-shaped micro pin fins (MCPF) was proposed, and the heat transfer and fluid flow characteristics of MCPF were numerically investigated. The geometric sizes optimization of MCPF reveals that there exist optimal fin sizes that satisfy both heat transfer and pressure drop conditions [25]. According to Stoddard’s research, which suggested that the stronger the bird flight ability, the more elongated the egg shape, which is deviated from the circular shape and closer to the streamlined shape. The elongated oval shape also reduces drag while ensuring that the egg has enough capacity, which helps the bird save more energy for flight. These conclusions were obtained by comparison of the egg shape of various birds and their flight ability [26]. Thus, in this study, oval-shaped micro pin fins are arranged in a smooth rectangular channel as a new finned microchannel, and the heat and heat transfer performance are investigated by numerical methods. Meanwhile, the influence of fin axial length ratio (*α*), fin width ratio (*β*), and fin height ratio (*γ*) on the flow and heat transfer performance is investigated, and the thermal enhancement factor is used to evaluate the overall performance of MOPF.

## 2. Model Description

**Figure 1:**
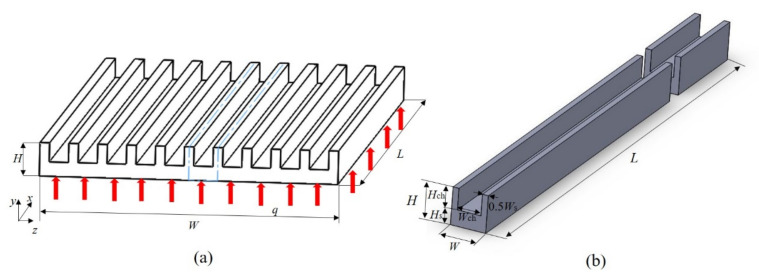
The geometric parameters of (**a**) rectangular MCHS and (**b**) a single MCHS.

**Figure 2:**
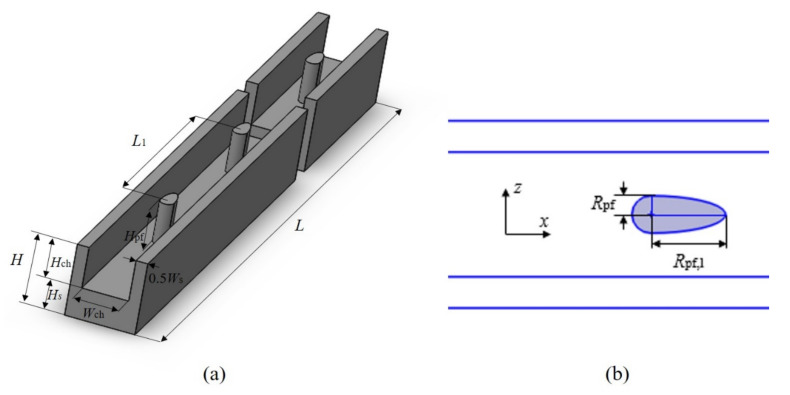
The geometric parameters of (**a**) a single MOPF and (**b**) oval-shaped micro pin fin.

A MCHS typically consists of several parallel channels, which are used to carry coolant fluid. The configuration of a typical MCHS is depicted schematically in Figure 1. The conventional rectangular microchannel is selected as a comparison. Because of the symmetry of microchannels, usually only one minimal unit of heat sink is simulated to perform the analysis, as illustrated in Figure 1b and Figure 2a. The newly proposed microchannel, which has oval-shaped micro pin fins, in a constant cross-section region in line is plotted in Figure 2. It has the same size with the corresponding rectangular microchannel except the inserted fins. Figure 2b is the top view of the channel with oval-shaped micro pin fin, which is composed of a semicircle and semi-ellipse. *R*_pf_ is the radius of the semicircle, which is also the length of a short half axis of the semi-ellipse. The length of long half axes of the semi-ellipse are denoted as *R*_pf,l_. The height of the micro pin fin is marked as *H*_pf,_ and its value is variable. In order to investigate the effects of geometric structure on fluid flow and heat transfer, three non-dimensional variables are defined as follows: *α* is defined as the ratio of the long semi-axial length (*R*_pf,l_) to the constant short semi-axial length (*R*_pf_) of the oval-shaped micro pin fin. The fin width ratio *β*(*β* = 2*R*_pf_/*W*_ch_) is defined as the ratio of the short axis length to the width of channel, and the fin height ratio (*γ* = *H*_pf_/*H*_ch_) is defined as the ratio of fin height to the channel height. The basic geometric parameters of a single microchannel are shown in Table 1.

## 3. Numerical Solution and Procedures

In this study, FLUENT 18.0 software was used to carry out three-dimensional numerical simulation calculation of flow and heat transfer characteristics of microchannels, and the calculation process was assumed as follows:

(1) The fluid flow in the channel is considered as three-dimensional steady laminar flow;
(2) The fluid in this work is water, which can be considered as incompressible Newtonian fluid. Meanwhile, the temperature variations are in the range of 293–320 K, therefore, the viscosity variation with temperature can be assumed as linear, and other solid and fluid property parameters are assumed as constant;
(3) The effects of gravity, volume force, surface tension, and thermal radiation are not taken into account.

### 3.1. Governing Equations

According to the above assumptions, the governing equations of the model are simplified as follows:

Mass equation:

(1) $$\frac{\partial u}{\partial x}+\frac{\partial v}{\partial y}+\frac{\partial w}{\partial z}=0$$

Momentum equation:

(2) $$u\frac{\partial u}{\partial x}+v\frac{\partial u}{\partial y}+w\frac{\partial u}{\partial z}=\frac{1}{\rho_{f}}\frac{\partial p}{\partial x}+\frac{\mu_{f}}{\rho_{f}}\left(\frac{\partial^{2}u}{\partial x^{2}}+\frac{\partial^{2}u}{\partial y^{2}}+\frac{\partial^{2}u}{\partial z^{2}}\right)$$

(3) $$u\frac{\partial v}{\partial x}+v\frac{\partial v}{\partial y}+w\frac{\partial v}{\partial z}=\frac{1}{\rho_{f}}\frac{\partial p}{\partial y}+\frac{\mu_{f}}{\rho_{f}}\left(\frac{\partial^{2}v}{\partial x^{2}}+\frac{\partial^{2}v}{\partial y^{2}}+\frac{\partial^{2}v}{\partial z^{2}}\right)$$

(4) $$u\frac{\partial w}{\partial x}+v\frac{\partial w}{\partial y}+w\frac{\partial w}{\partial z}=\frac{1}{\rho_{f}}\frac{\partial p}{\partial z}+\frac{\mu_{f}}{\rho_{f}}\left(\frac{\partial^{2}w}{\partial x^{2}}+\frac{\partial^{2}w}{\partial y^{2}}+\frac{\partial^{2}w}{\partial z^{2}}\right)$$

Energy equation (for the fluid):

(5) $$u\frac{\partial T_{f}}{\partial x}+v\frac{\partial T_{f}}{\partial y}+w\frac{\partial T_{f}}{\partial z}=\frac{k_{f}}{\rho_{f}c_{\mathrm{pf}}}\left(\frac{\partial^{2}T_{f}}{\partial x^{2}}+\frac{\partial^{2}T_{f}}{\partial y^{2}}+\frac{\partial^{2}T_{f}}{\partial z^{2}}\right)$$

Energy equation (for the substrate conduction):

(6) $$0=k_{s}\left(\frac{\partial^{2}T_{s}}{\partial x^{2}}+\frac{\partial^{2}T_{s}}{\partial y^{2}}+\frac{\partial^{2}T_{s}}{\partial z^{2}}\right)$$

where *u*, *v*, and *w* are respectively the velocity component in the *x*, *y*, and *z* directions; *ρ*_f_, *μ*_f_, *c*_pf_, *k*_f_, and *k*_s_ are density, dynamic viscosity, specific heat capacity, and thermal conductivity of liquid and solid, respectively. *P*, *T*_f,_ and *T*_s_ are the pressure and temperature of liquid and solid, respectively.

### 3.2. Boundary Conditions

(1) The channel inlet is set as the velocity inlet boundary condition, and the fluid inlet temperature is constant at 293 K;

(7) $$x=0,u_{f}=u_{\mathrm{in}};T_{f}=T_{\mathrm{in}}=293K$$

(2) At the channel outlet, the pressure-outlet boundary is applied;

(8) $$x=L,P_{f}=P_{\mathrm{out}}=0$$

(3) The solid walls coupled with fluid are set as the coupled boundary condition, and the no-slip condition boundary is applied on the fluid–solid interface.

(9) $$-k_{s}{\left(\frac{\partial T_{s}}{\partial n}\right)}_{w}=-k_{s}\left(\frac{\partial T_{f}}{\partial n}\right),u=v=w=0$$

(4) The symmetry condition is used in two side walls of the microchannel;

(10) $$\frac{\partial T_{s}}{\partial y}=0,\;(y=0,\;y=0.15\;\mathrm{mm})$$

(5) The bottom surface of the microchannel is set as the uniform heat flux boundary, and the top surface of the channel is adiabatic;

(11) $$z=0,q=10^{6}W/m^{2};z=H,{\left(\frac{\partial T}{\partial n}\right)}_{w}=0$$

The governing equations are solved using the finite volume-based computational fluid dynamics solver FLUENT. The SIMPLEC algorithm (*p*-*v* coupling) is applied to solve the governing equations. Second order discretization scheme is adopted for the pressure equation, and second-order upwind scheme is employed for discretization of momentum and energy equations. The residual criteria of continuity equation and velocity are set as 10^−6^ and that of energy equation is 10^−7^. 

### 3.3. Data Reduction

The inlet Reynolds number is defined as:

(12) $$Re=\frac{\rho u_{\mathrm{in}}D_{h}}{\mu }$$

where *u*_in_ is the channel inlet velocity, *ρ* is the water density, and *D*_h_ is the hydraulic diameter of the microchannel, which can be expressed as:

(13) $$D_{h}=\frac{2W_{\mathrm{ch}}H_{\mathrm{ch}}}{W_{\mathrm{ch}}+H_{\mathrm{ch}}}$$

The apparent friction coefficient of the microchannel can be expressed as:

(14) $$f_{\mathrm{app},\mathrm{ave}}=\frac{2\Delta pD_{h}}{\rho Lu_{m}^{2}}$$

where Δ*p* is the pressure drop of microchannel, *L* is the channel length, and *u*_m_ is the average velocity.

The average convective heat transfer coefficient is written as:

(15) $$h_{\mathrm{ave}}=\frac{Q}{A_{\mathrm{con}}\Delta T}=\frac{qA_{b}}{A_{\mathrm{con}}\left(T_{b}-T_{f,m}\right)}$$

And the Nusselt number of microchannels are given as follows:

(16) $$Nu_{\mathrm{ave}}=\frac{h_{\mathrm{ave}}D_{h}}{k_{f}}$$

where *q* is the constant heat flux, *A*_b_ is the area of channel bottom wall; *T*_b_ and *T*_f,m_ are average bottom wall temperature and average fluid temperature respectively; *A*_con_ is the effective heat transfer area; and *k*_f_ is fluid thermal conductivity.

In order to evaluate the overall performance of the microchannel with oval-shaped micro pin fins, the thermal enhancement factor *η* was introduced to measure the heat transfer enhancement against the pressure drop increment for laminar flow with chaotic advection. The bigger *η* is, the better the overall performance of the microchannel. The expression for *η* is given by [27]

(17) $$\eta =\frac{Nu_{\mathrm{ave}}/Nu_{0}}{{\left(f_{\mathrm{ave}}/f_{0}\right)}^{1/3}}$$

where *Nu*_0_ and *f*_0_ are the average Nusselt number and average apparent friction coefficient of the rectangular microchannel, respectively.

### 3.4. Grid Independence and CFD Simulations

**Figure 3:**
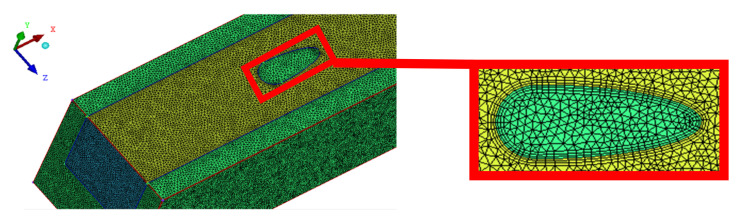
Computational grid in the x-z plane for MOPF (*y* = 0.25 mm).

The ICEM meshing software was utilized to generate meshes for the solver, and the unstructured meshing method was adopted. Figure 3 shows the local grid division diagram of a microchannel with oval-shaped pin fin (*α* = 3, *β* = 0.3, *γ* = 1) in the *x*-*z* plane (*y* = 0.25 mm). The green region is the calculation domain of solid silicon, and the yellow region is the calculation domain of deionized water. For every microchannel heat sink, a grid independence test is conducted using several different mesh sizes. The average Nusselt number *Nu*_ave_ and pressure drop Δ*p* were selected as parameters for grid independent verification. Meanwhile, the relative error between the finest grids (*J*_1_) and other grids (*J*_2_) is written as:

(18) $$e\%=\frac{\left|J_{2}-J_{1}\right|}{J_{1}}\times 100\%$$

By gradually refining the meshes, five different grid quantities (856,372 cells, 1,626,235 cells, 2,235,843 cells, 2,806,421 cells, 3,414,287 cells) were selected for verification, and the results were shown in Table 2. The relative error *e*% is less than 2% when the number of meshes is 2,235,843 cells. In order to reduce the computation time, 2,235,843 cell grids were selected for the subsequent simulation calculation.

## 4. Results and Discussion

### 4.1. Verification of Numerical Models

It is necessary to check the accuracy and reliability of the mathematical model; numerical results of friction factor and local Nusselt number for the rectangular MCHS are compared with the theoretical solutions. The relation of apparent friction coefficient under the condition of fully developed laminar flow can be expressed as:

(19) $$f=\frac{Po}{Re}$$

According to the model proposed by Shah and London [28], Poiseuille number can be written as follows:

(20) $$Po=96\left[1-1.3553\left(\mathrm{AR}\right)+1.9467{\left(\mathrm{AR}\right)}^{2}-1.7012{\left(\mathrm{AR}\right)}^{3}+0.9564{\left(\mathrm{AR}\right)}^{4}-0.2537\right]$$

The relationship between apparent friction coefficient and Reynolds number and Poiseuille number is shown as follows:

(21) fapp,aveRe=3.2L/ReDh2+Po2

where AR is the aspect ratio of the microchannel, which is 1 in this study; 

The local Nusselt number of the three sides-heated microchannel is written as [29]:

(22) $$Nu_{3,x}=Nu_{4,x}\left(Nu_{3,\infty }/Nu_{4,\infty }\right)$$

where *Nu*_3,∞_ and *Nu*_4,∞_ represent fully developed Nusselt numbers for three-side heating and four-side heating, respectively. The local Nusselt number *Nu*_4,*x*_ is calculated by Kandlikar model as follows [30]:

(23) $$Nu_{4,x}=6.7702-3.1702x*+0.4187{\left(\ln x*\right)}^{2}+2.1555\ln x*+2.76\times 10^{{}^{-6}}{\left(x*\right)}^{-1.5}$$

where *x* is the distance to the inlet, and x* is dimensionless axial distance, which can be expressed as:

(24) $$x*=\frac{x}{RePrD_{h}}$$

(25) $$Pr=\frac{\mu C_{p}}{k}$$

The variation of the pressure drop characteristics of the microchannel can be verified by the following formula [31]:

(26) $$\Delta p=\frac{2\left(fRe\right)\mu u_{m}L}{D_{h}}+\frac{a\left(x\right)\rho u_{m}^{2}}{2}$$

The *a*(*x*) formula is given as follows:

(27) $$\begin{array}{l} a\left(x\right)=0.6796+1.2197\left(\mathrm{AR}\right)+3.3089\left({\mathrm{AR}}^{2}\right)-9.5921\left({\mathrm{AR}}^{3}\right)+\\ 8.9089\left({\mathrm{AR}}^{4}\right)-2.9959\left({\mathrm{AR}}^{5}\right) \end{array}$$

**Figure 4:**
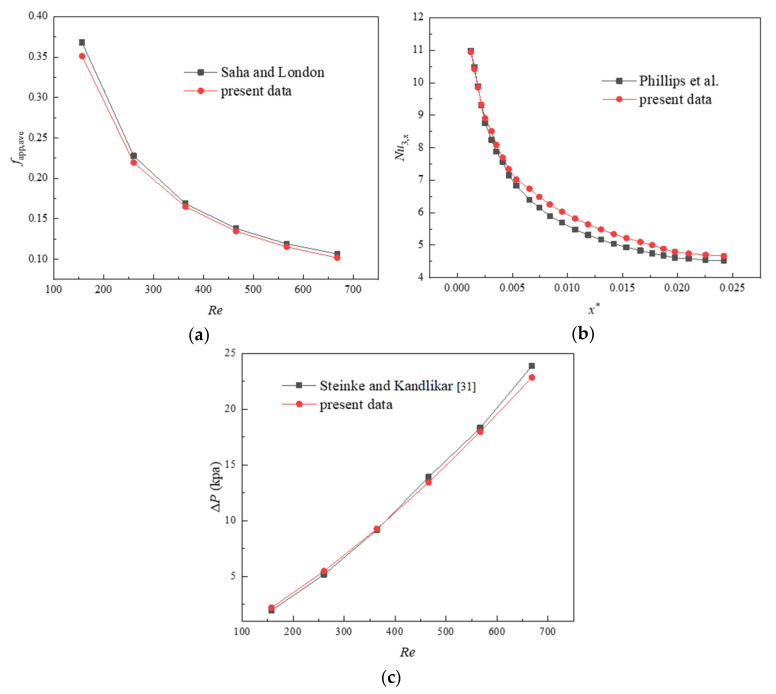
Numerical validation: (**a**) apparent friction factor *f*_app,ave_ (**b**) local Nusselt number *Nu*_4,*x*_ along the flow direction, and (**c**) pressure drop.

Figure 4 shows the comparison results between the numerical calculation and the references. As can be seen from the figure, the maximum deviation of *f*_app,ave_ and *Nu*_3,*x*_ according to the above formula are 3.8% and 4.01%, respectively. The numerical results of pressure drop are in good agreement with the theoretical data at all given *Re*, and deviation is within 3%. Therefore, the numerical method adopted in this paper is accurate and effective.

### 4.2. Thermo/Hydraulic Optimization of Fin Axial Length Ratio (α)

**Figure 5:**
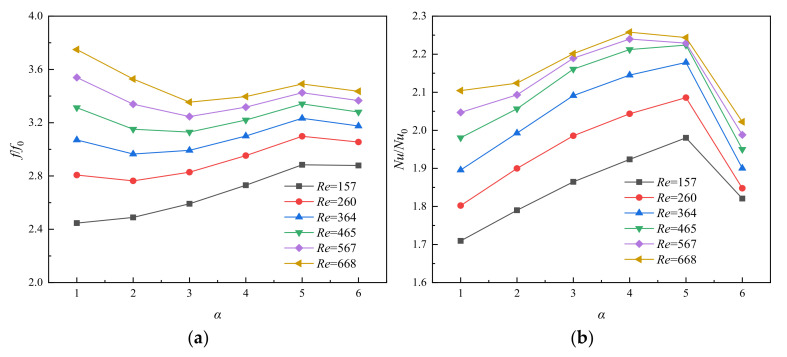
Relationship between (**a**) *f*/*f*_0_, (**b**) *Nu*/*Nu*_0_, and *α* for varying *Re*, *β* = 0.3, *γ* = 1.

The relationship between *f*/*f*_0_ and *α* (=*R*_pf,l_/*R*_pf_) under different *Re* is shown in Figure 5a, of which *f*_0_ represents the average apparent friction coefficient of the rectangular microchannel. As can be seen from Figure 5a, the value of *f*/*f*_0_ increases obviously with the increasing of *Re*. Furthermore, according to calculation results, the values of *f*/*f*_0_ as a function of *α* are found to: first increase, then decrease and increase again, except for the condition of *Re* = 157. This phenomenon is thought to be largely determined by the combined effects of friction resistance and pressure drag. They are caused by the viscous effect, which induces the surface frictional force and flow separation and vortex shedding. 

**Figure 6:**
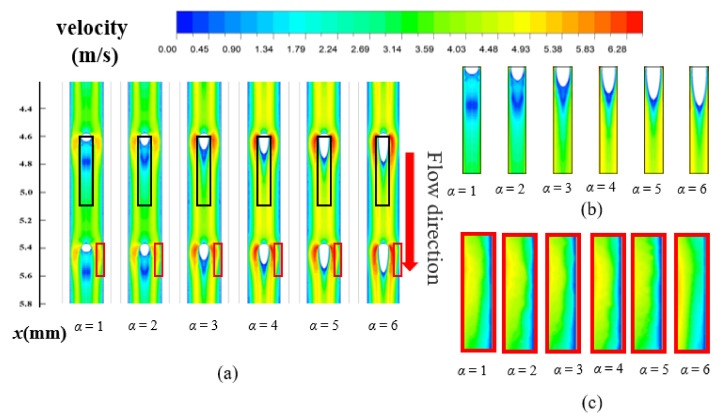
(**a**) The velocity distribution along the horizontal plane (*y* = 0.25 mm), (**b**) The partial enlarged drawing of fin tail, (**c**) The partial enlarged drawing of side walls for MOPF with different shapes (*α* = 1~6) of fin at *Re* = 668.

Figure 6 represents the velocity distribution along the horizontal plane (z = 0.25 mm) for MOPF with different *α*, and the drawings of partial enlargement are displayed at the same time. As can be seen from the figure, placing oval-shaped pin fins in flow path generates boundary layer separation and a wake region is formed in the downstream of the fin. From Figure 6b, it is easy to observe, the larger the value of *α*, the smaller wake region, which indicates that the pressure drag has been effectively suppressed, and this is attributed to the shape of the fins. However, due to the expansion of fin surface area, the frictional resistance inevitably increases. Thus, the total resistance of the microchannel is the result of the superposition of these two. However, when the value of *α* continues to increase, the increased amplitude of friction resistance is smaller than the decreased amplitude of pressure drag, perhaps as a consequence, the value of *f*/*f*_0_ decreases at *α* = 6.

The relationship between *Nu*/*Nu*_0_ and *α* (=*R*_pf,l_/*R*_pf_) in different *Re* is shown in Figure 5b, and *Nu*_0_ represents the Nusselt number of the Rec microchannel. It can be seen from Figure 5b that the value of *Nu*/*Nu*_0_ generally increases firstly and then decreases with the increase of *α* at the same *Re*. MOPF with *α* = 5 has the maximum value of *Nu*/*Nu*_0_ for *Re* ≤ 465; but MOPF with *α* = 4 yields the highest value of *Nu*/*Nu*_0_ for *Re* > 465. Inserting pin fins into the heat sink channel increases the heat transfer area, thus improving the convection process. On the other hand, the velocity distribution is another determining factor to convective heat transfer mechanism. Figure 6 shows the velocity distribution along the horizontal plane for MOPF with different fin axial length ratio (*α*) at *Re* = 668, and (b), (c) is the partial enlarged detail of fluid velocity at the side wall of the fins passage. It suggests that the enhanced heat transfer of the microchannel with micro pin fins is primarily attributed to flow separation, disturbance effect, and the vortexes in the mainstream rather than surface area enlargement. However, as the value of *α* increases, the cross-section shape of the micro pin fin on the y-z plane is closer to streamline, which causes the separation point of the boundary layer to move backward and the wake vortex region becomes smaller. Meanwhile, the impact of fluid on the wall is reduced because of the streamline fins, which leads to the weakening of the fluid disturbance. Thus, the overall heat transfer performance of the microchannel decreases for the higher value of *α*.

**Figure 7:**
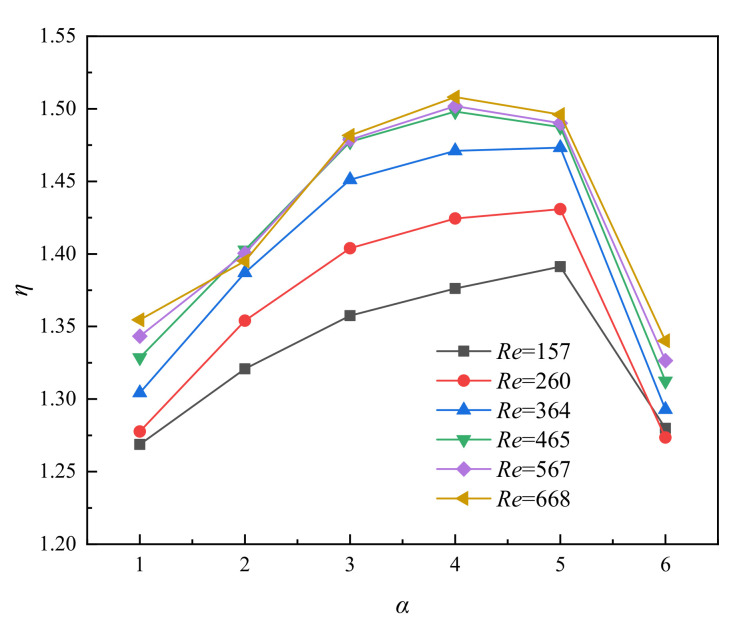
Relationship between *η* and *α* for varying *Re*, *β* = 0.3, *γ* = 1.

The improvement of heat transfer performance often leads to the increase of flow resistance, which certainly leads to increase the consumption of pump power. Therefore, it is necessary to comprehensively evaluate the increasement of heat transfer performance and flow resistance. According to the reasons mentioned above, the thermal enhancement factor *η* is introduced to compare the comprehensive performance of MOPF as shown in Equation (12). Figure 7 depicted that the value of *η* increases firstly and then decreases with the increase of *α*. The MOPF with *α* = 5 has the best comprehensive performance for Re ≤ 364, and the highest overall performance for *Re* > 364 is *α* = 4, and *η* reaches a maximum value of 1.52 when *Re* = 668. In addition, the value of *η* is affected slightly by the *Re*. 

### 4.3. Thermo/Hydraulic Optimization of Fin width Ratio (β)

**Figure 8:**
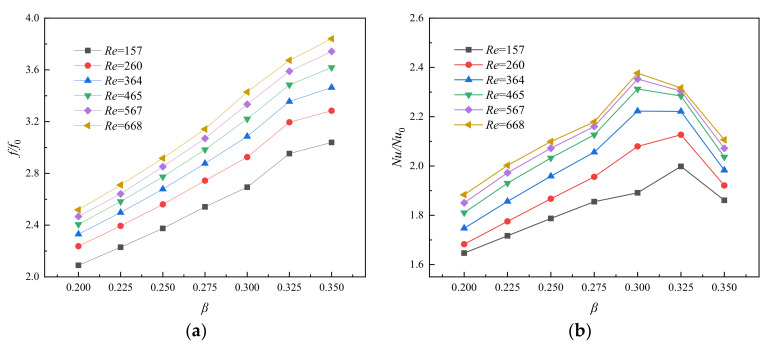
Relationship between (**a**) *f*/*f*_0_, (**b**) *Nu*/*Nu*_0_, and *β* for varying *Re*, *α* = 4, *γ* = 1.

After optimizing the shape of the micro pin fin, the second design parameter, which is the fin width ratio *β (*=2*R*_pf_/*W*_ch_), is also examined here. As shown in Figure 8a, *f*/*f*_0_ presents a gradual upward trend with the increase of the fin width ratio *β* in the passage, and an approximate linear increase was found over a large Reynolds number range. The reasons for this result can be explained from the following three aspects. Firstly, the blocking sections of the front of the fins increase because of the wider fin width, which results in a large blocking effect. Secondly, as the width of the fins increases, the contact area between the fluid and the solid increases, and accordingly, the friction resistance also increases. Finally, with the increase of the width of the fins, the distance between the outer side of the fins and the side wall of the channel becomes narrower, which results in a significant increase in the flow rate of fluid through these areas. The separation point of the boundary layer moves forward, and a large low-pressure circulation zone is formed at the tail of the fins, which further increases the pressure drag of fins. 

**Figure 9:**
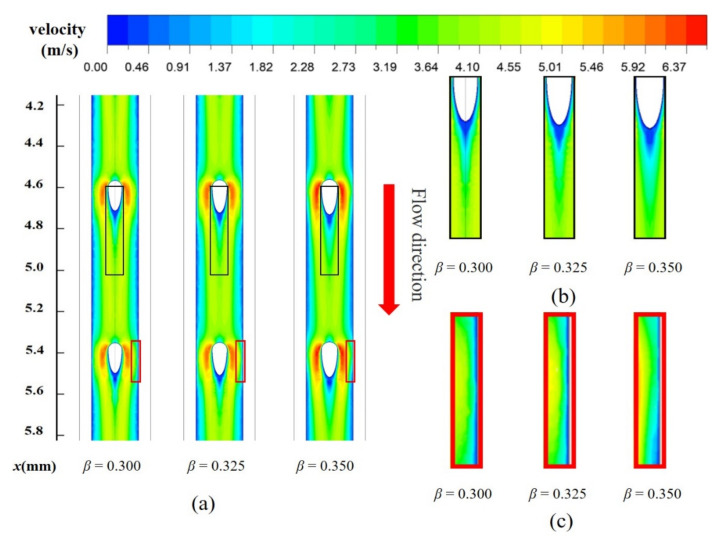
(**a**) The velocity distribution along the horizontal plane (*y*= 0.25 mm), (**b**) The partial enlarged drawing of fin tail, (**c**) The partial enlarged drawing of side walls for MOPF with relative fin diameter (*β* = 0.300~0.325) at *Re* = 668.

In this section, the effect of fin width ratio *β* on heat transfer performance of MOPF is investigated. Figure 8b illustrates the *Nu*/*Nu*_0_ as a function of the microchannel fin width ratio *β* at different *Re*. It is noted that the *Nu*/*Nu*_0_ firstly increases and then decreases with the increase of the fin width ratio *β*. The heat transfer area increases with the increase of the fin width, and the heat transfer performance is significantly enhanced. In addition, the flow rate between the channel wall and fin increases because of the reduction of circulation area. However, as the size of fins increases, the area of trailing vortex area increases, which resulting in the heat transfer performance degradation. Figure 9 shows the velocity contours and its partial enlargement along the horizontal plane (*z* = 0.25 mm) for MOPF with relative fin diameter (*β* = 0.300~0.325) of fin at *Re* = 346. It is evident from the picture that the trailing vortex region becomes larger with the increase of fin diameter. Meanwhile, because of the longer fin tail, the scouring effect of the fluid on the side wall of the channel is weakened, and the boundary layer of the fluid on the side wall becomes thicker. Thus, the overall heat transfer performance is weakened.

Besides, it should not be neglected that the peak value of *Nu*/*Nu*_0_ corresponds to different width ratio values within different Reynolds number ranges. When *Re* is lower than 364, *Nu*/*Nu*_0_ reaches its peak value when *β* = 0.325, but the peak point of *Nu*/*Nu*_0_ moves left to *β* = 0.300 when Re ≥ 364. This result implies that the increase of flow velocity causes premature separation of the boundary layer at smaller fin width, which increases the wake vortex region and weakens the thermal performance.

**Figure 10:**
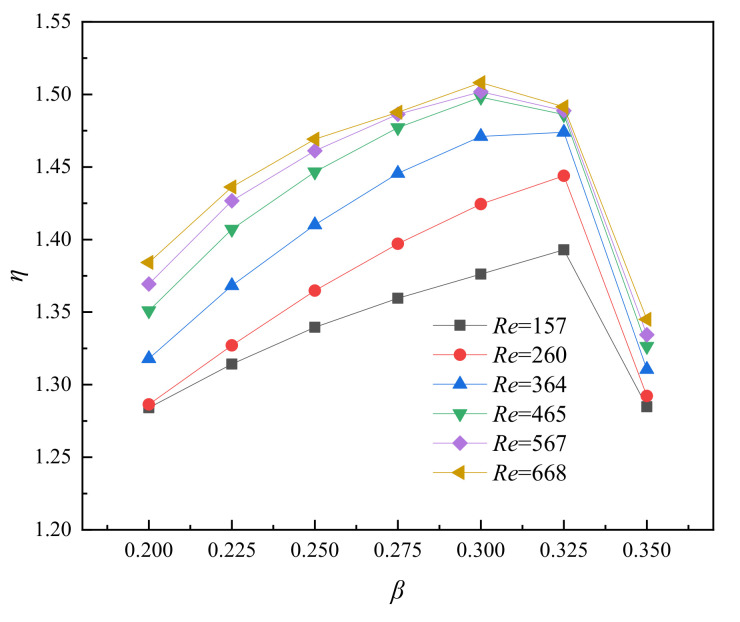
Relationship between *η* and *β* for varying *Re*, *α* = 4, *γ* = 1.

By comparing Figure 8a,b it is found that the change of *f*/*f*_0_ is not always positively correlated with the change of *Nu*/*Nu*_0_. The former is positively correlated with *β*, but *Nu*/*Nu*_0_ shows a large decrease in the range of *β* = 0.325–0.350. In order to comprehensively reflect the influence of relative fin width on channel performance, the variation relationship of the *η* with the width ratio *β* at different *Re* is shown in Figure 10. The curve generally shows the same trend as *Nu*/*Nu*_0_. In the range of low *Re* (<346), *β* = 0.325 has a better comprehensive performance, but the best *η* in the range of high *Re* (>346) occurs in *β* = 0.300.

### 4.4. Thermo/Hydraulic Optimization of Fin Height Ratio (γ)

**Figure 11:**
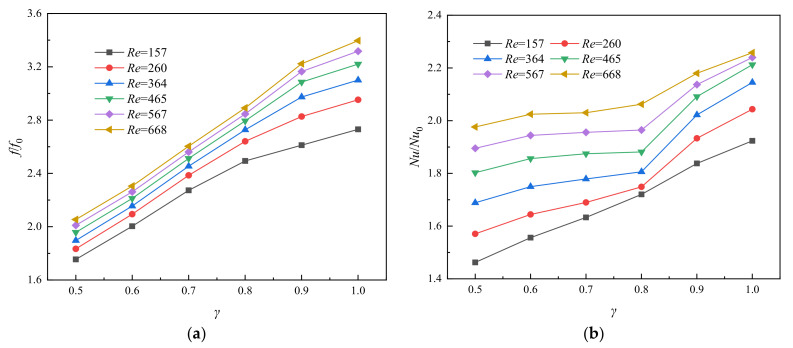
Relationship between (**a**) *f*/*f*_0_, (**b**) *Nu*/*Nu*_0_, and *γ* for varying *Re*, *α* = 4, *β* = 0.3.

Except for the shape and size of fins, the height of the fin also has a great influence on flow and heat transfer performance of the microchannel. The height of fins not only affects the heat transfer area, but also affects the distribution of fluid velocity and increases fluid disturbance. The results of *f*/*f*_0_ and *Nu/Nu*_0_ for variable *fin height ratio* (*γ* = *H*_pf_/*H*_ch_) with constant values of *α* = 4 and *β* = 0.3 are gained and described in Figure 11. As can be seen from Figure 11a, the value of *f*/*f*_0_ increases with the increase of *Re* at the same relative fin height, and it was positively correlated with the relative fin height under the same *Re* number condition. The change of *f*/*f*_0_ can be attributed to two aspects: As the height of fin increases, the shear force on the fluid–solid interface and the pressure drag increase accordingly.

**Figure 12:**
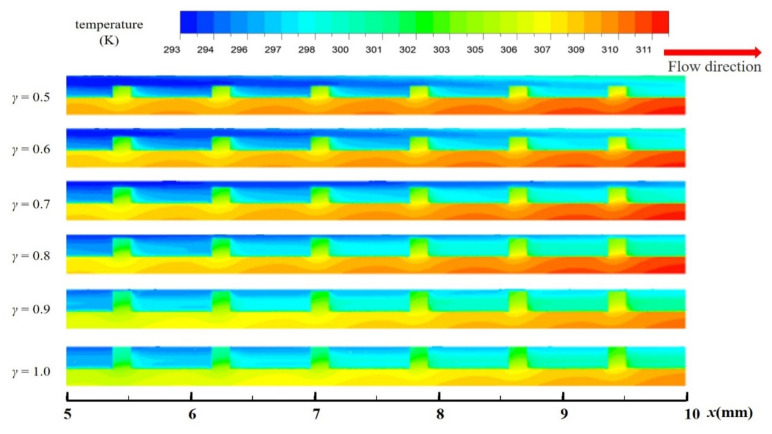
The temperature distribution along the central plane (*y* = 0 mm) for MOPF with different values of *γ*, *Re* = 567.

**Figure 13:**
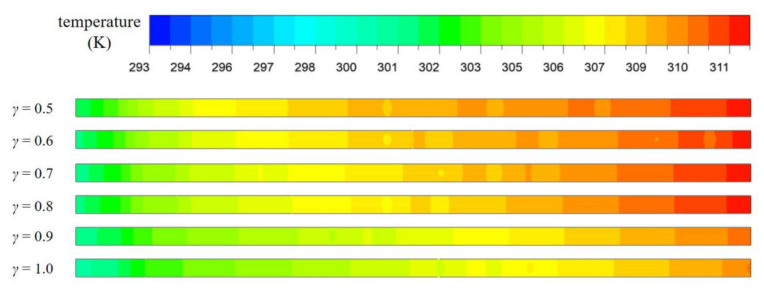
Temperature distribution on the heated bottom wall with relative fin height, *Re* = 567.

Figure 11b shows the variation of *Nu*/*Nu*_0_ with *γ* under different Reynolds Numbers. The value of *Nu*/*Nu*_0_ increases with the increase of *γ*, indicating that the heat transfer performance of the channel is gradually improved by the increase of the fin height. This is the result of the combined effect of increasement of heat transfer area and fluid disturbance. The temperature distribution of axial surface (*y* = 0 mm) in the second half of the microchannel at *Re* = 567 was observed in Figure 12. The average temperature of fluid is the highest when *γ* = 1.0, while the average temperature of solid area is the lowest. This phenomenon is also reflected in Figure 13. It represents the distribution of temperature gradient on the heated bottom wall corresponding to different fin height at the same inlet *Re*. From the figure, it is worth noting that the temperature of the bottom wall decreases with the increase of the relative fin height, and the temperature is well-distributed when the relative fin height is aequal to 1. Such phenomenon is in agreement with a previous study reported by Jia et al. [24]

**Figure 14:**
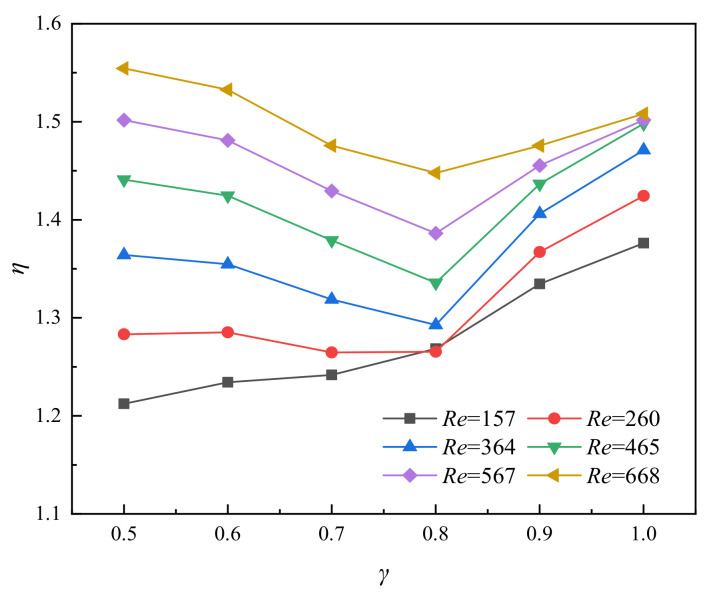
Relationship between *η* and *γ* for varying *Re*, *α* = 4, *β* = 0.3.

As stated above, in addition to the increase in thermal performance, significance also increases with pressure drop. Thus, the comprehensive performance of flow and heat transfer is necessary to obtain and compare. The variation of the thermal enhancement factor *η* with *Re* is obtained as illustrated in Figure 14. The value of *η* increases with the increase of *Re*, while the value of *η* decreases first and then increases with the increase of the value of *γ* at the same *Re*. The value of *η* is the highest when *γ* = 0.5 and *Re* = 668, while the value of *η* is the lowest when *Re* = 157.

## 5. Conclusions

In this paper, a new type of microchannel heat sink with oval-shaped pin fins arranged uniformly inside the microchannel is proposed. The flow and heat transfer characteristics of the fluid at the channel are numerically investigated. The influences of the fin axial length ratio (*α*), width ratio (*β*), and height ratio (*γ*) on the overall flow heat transfer performance of the microchannel are respectively studied. The specific conclusions are as follows:

(1) The oval-shaped micro pin fins are helpful to heat transfer enhancement due to flow separation, disturbance effect, and the vortexes in the mainstream. Compared to the smooth rectangular microchannel, the microchannel proposed in the present paper shows more uniform and lower temperature at the substrate of the heat sink and gets a better heat transfer performance.
(2) In the range of parameters in this study, the friction factor increases as the fin width and height increases. However, with the increase of the fin axial length ratio, the friction factor first increases, then decreases and increases again, which suggests that extending the fin tail and making it closer to the streamlined will reduce the pump power to some extent.
(3) With the increase of fin axial length and width ratio, the Nusselt number increases at first and then increases slightly, meanwhile, the thermal enhancement factor *η* shows a similar trend. At the high *Re* range, the MOPF with *α* = 4, *β* = 0.3, and *γ* = 1 has the best comprehensive performance, while at the low *Re* range, the MOPF with *α* = 5, *β* = 0.325, and *γ* = 1 shows the best overall performance.

## Figures and Tables

**Table 1:** Geometric characteristics of MOPF.

No.	Parameter	Value	No.	Parameter	Value
1	*H*	0.35 mm	6	*L*_1_	0.8 mm
2	*H*_ch_	0.2 mm	7	*W*_ch_	0.2 mm
3	*H*_s_	0.15 mm	8	*W*_sw_	0.05 mm
4	*H*_pf_	0.1–0.2 mm	9	*R*_pf_	0.02–0.08 mm
5	*L*	10 mm	10	*R*_pf,l_	0.03–0.18 mm

**Table 2:** Grid independence test.

Grid Numbers × 10^6^	*Nu*	*e*%	Δ*p* (Pa)	*e*%
0.8563	11.10	4.86	16,784.23	5.06
1.6262	11.34	2.64	16,489.56	3.37
2.2358	11.57	0.95	16,096.45	1.01
2.8064	11.62	0.43	16,024.39	0.59
3.4142	11.64		15,934.27	

## Data Availability

Not applicable.

## Nomenclature

*A*_con_	Convection heat transfer area (m^2^)	*Re*	Reynolds number
*A*_b_	heating area (m^2^)	*T*_f,m_	average temperature of fluid (K)
*A*_b,pf_	bottom area of micro pin fin (m^2^)	Δ*T*	temperature difference (K)
*A*_ch_	channel bottom surface area (m^2^)	*u*,*v*,*w*	velocity component in the x, *y* and *z* direction
AR	aspect ratio	*W*	width of MCHS (m)
*c*_p_	specific heat capacity (J/(kg·K))	*W*_ch_	channel width (m)
*D*_h_	hydrodynamic diameter (m)	*W*_sw_	side wall width (m)
*D*_pf_	the diameter of micro pin fin (m)	*Greek symbols*	
*e*	relative error	*α*	fin axis length ratio
*f*	friction factor	*β*	fin width ratio
*h*	heat transfer coefficient (W/(m^2^·K))	*γ*	fin height ratio
*H*	height of the MCHS (m)	*η*	thermal enhancement factor
*H*_ch_	height of microchannel (m)	*μ*	dynamic viscosity (Pa·s)
*H*_pf_	height of the micro pin fin (m)	*ρ*	density (kg/m^3^)
*H*_s_	height of the substrate (m)	*Subscript*	
*k*	thermal conductivity (W/(m·K))	app	apparent
*L*	length of the microchannel (m)	ave	average
*L*_1_	space between micro pin fins (m)	b	baseplate
*Nu*	Nusselt number	f	fluid
*N*_pf_	number of micro pin fins	in	inlet
*pp*	pump power (W)	m	mean
Δ*p*	pressure drop (Pa)	out	outlet
*Pr*	Prandtl number	pf	micro pin fin
*Po*	Poiseuille number	s	solid
*q*	heat flux (W/m^2^)	sw	side wall of the microchannel
*Q*	total heat input (W)	w	wall

## References

[1] Ndao S., Peles Y., Jensen M.K. (2009). Multi-objective thermal design optimization and comparative analysis of electronics cooling technologies. Int. J. Heat Mass Transf..

[2] Karathanassis I., Papanicolaou E., Belessiotis V., Bergeles G. (2013). Multi-objective design optimization of a micro heat sink for Concentrating Photovoltaic/Thermal (CPVT) systems using a genetic algorithm. Appl. Therm. Eng..

[3] Noh M., Hazwani N., Sidik N.A.C. (2014). Numerical Simulation of Nanofluids for Improved Cooling Efficiency in Microchannel Heat Sink. Appl. Mech. Mater..

[4] Lu Y.W., Kandlikar S.G. (2011). Nanoscale Surface Modification Techniques for Pool Boiling Enhancement—A Critical Review and Future Directions. Heat Transf. Eng..

[5] Deng D., Pi G., Zhang W., Wang P., Fu T. (2019). Numerical Study of Double-Layered Microchannel Heat Sinks with Different Cross-Sectional Shapes. Entropy.

[6] Gunnasegaran P., Mohammed H.A., Shuaib N.H., Saidur R. (2010). The effect of geometrical parameters on heat transfer characteristics of microchannels heat sink with different shapes. Int. Commun. Heat Mass Transf..

[7] Lin L., Zhao J., Lu G., Wang X.D., Yan W.M. (2017). Heat transfer enhancement in microchannel heat sink by wavy channel with changing wavelength/amplitude. Int. J. Therm. Sci..

[8] Ermagan H., Rafee R. (2018). Numerical investigation into the thermo-fluid performance of wavy microchannels with superhydrophobic walls. Int. J. Therm. Sci..

[9] Toghraie D., Abdollah M.M.D., Pourfattah F., Akbari O.A., Ruhani B. (2018). Numerical investigation of flow and heat transfer characteristics in smooth, sinusoidal and zigzag-shaped microchannel with and without nanofluid. J. Therm. Anal. Calorim..

[10] Srivastava P., Dewan A. (2018). Effect of bifurcation on thermal characteristics of convergent-divergent shaped microchannel. ASME J. Therm. Sci. Eng. Appl..

[11] Li Y.F., Xia G.D., Ma D.D., Jia Y.T., Wang J. (2016). Characteristics of laminar flow and heat transfer in microchannel heat sink with triangular cavities and rectangular ribs. Int. J. Heat Mass Transf..

[12] Zhai Y.L., Xia G.D., Liu X.F., Li Y.F. (2014). Heat transfer in the microchannels with fan-shaped reentrant cavities and different ribs based on field synergy principle and entropy generation analysis. Int. J. Heat Mass Transf..

[13] Datta A., Sharma V., Sanyal D., Das P. (2019). A conjugate heat transfer analysis of performance for rectangular microchannel with trapezoidal cavities and ribs. Int. J. Therm. Sci..

[14] Wang R., Wang W., Wang J., Zhu Z. (2018). Optimization of Trapezoidal Grooved Microchannel Heat Sink Using Nanofluids in a Micro Solar Cell. Entropy.

[15] Croce G., D’agaro P., Nonino C. (2007). Three-dimensional roughness effect on microchannel heat transfer and pressure drop. Int. J. Heat Mass Transf..

[16] Ghani I.A., Sidik N.A.C., Mamat R., Najafi G., Ken T.L., Asako Y., Japar W.M.A.A. (2017). Heat transfer enhancement in microchannel heat sink using hybrid technique of ribs and secondary channels. Int. J. Heat Mass Transf..

[17] Shi X., Li S., Mu Y., Yin B. (2019). Geometry parameters optimization for a microchannel heat sink with secondary flow channel. Int. Commun. Heat Mass Transf..

[18] Ali N., Bahman A.M., Aljuwayhel N.F., Ebrahim S.A., Mukherjee S., Alsayegh A. (2021). Carbon-Based Nanofluids and Their Advances towards Heat Transfer Applications—A Review. Nanomaterials.

[19] He Z., Yan Y., Zhang Z. (2021). Thermal management and temperature uniformity enhancement of electronic devices by micro heat sinks, A review. Energy.

[20] Vasilev M.P., Abiev R.S., Kumar R. (2021). Effect of circular pin-fins geometry and their arrangement on heat transfer performance for laminar flow in microchannel heat sink. Int. J. Therm. Sci..

[21] Vilarrubí M., Riera S., Ibañez M., Omri M., Laguna G., Frechette L., Barrau J. (2018). Experimental and numerical study of micro-pin-fin heat sinks with variable density for increased temperature uniformity. Int. J. Therm. Sci..

[22] Prajapati Y.K. (2019). Influence of fin height on heat transfer and fluid flow characteristics of rectangular microchannel heat sink. Int. J. Heat Mass Transf..

[23] Ventola L., Dialameh M., Fasano M., Chiavazzo E., Asinari P. (2016). Convective heat transfer enhancement by diamond shaped micro-protruded patterns for heat sinks: Thermal fluid dynamic investigation and novel optimization methodology. Appl. Therm. Eng..

[24] Wang X., Jiang H. (2018). Design of origami fin for heat dissipation enhancement. Appl. Therm. Eng..

[25] Jia Y., Xia G., Li Y., Ma D., Cai B. (2018). Heat transfer and fluid flow characteristics of combined microchannel with cone-shaped micro pin fins. Int. Commun..

[26] Stoddard M.C., Yong E.H., Akkaynak D., Sheard C., Tobias J.A., Mahadevan L. (2017). Avian egg shape, Form, function, and evolution. Science.

[27] Karwa R., Sharma C., Karwa N. (2013). Performance Evaluation Criterion at Equal Pumping Power for Enhanced Performance Heat Transfer Surfaces. J. Sol. Energy.

[28] Shah R.K., London A.L. (1978). Laminar Flow Forces Convection in Ducts.

[29] Phillips R.J. (1987). Microchannel Heat Sinks. Ph.D. Thesis.

[30] Kandlikar S., Garimella S., Li D., Colin S., King M.R. (2006). Heat Transfer and Fluid Flow in Minichannels and Microchannels.

[31] Steinke M.E., Kandlikar S.G. (2006). Single-phase liquid friction factors in microchannels. Int. J. Therm. Sci..

